# Transcriptome analyses of early cucumber fruit growth identifies distinct gene modules associated with phases of development

**DOI:** 10.1186/1471-2164-13-518

**Published:** 2012-10-02

**Authors:** Kaori Ando, Kevin M Carr, Rebecca Grumet

**Affiliations:** 1 Program in Plant Breeding, Genetics and Biotechnology, Michigan State University, East Lansing, MI, 48824, USA; 2 Research Technology Support Facility, Michigan State University, East Lansing, MI, 48824, USA; 3 Department of Horticulture and Program in Plant Breeding, Genetics and Biotechnology, Michigan State University, East Lansing, MI, 48824, USA; 4 Present address: Department of Crop and Soil Science, Washington State University, Pullman, WA, 99164, USA

**Keywords:** *Cucumis sativus*, Exponential fruit growth, Fruit maturation, Fruit set, Fruit surface, Gene expression

## Abstract

**Background:**

Early stages of fruit development from initial set through exponential growth are critical determinants of size and yield, however, there has been little detailed analysis of this phase of development. In this study we combined morphological analysis with 454 pyrosequencing to study transcript level changes occurring in young cucumber fruit at five ages from anthesis through the end of exponential growth.

**Results:**

The fruit samples produced 1.13 million ESTs which were assembled into 27,859 contigs with a mean length of 834 base pairs and a mean of 67 reads per contig. All contigs were mapped to the cucumber genome. Principal component analysis separated the fruit ages into three groups corresponding with cell division/pre-exponential growth (0 and 4 days post pollination (dpp)), peak exponential expansion (8dpp), and late/post-exponential expansion stages of growth (12 and 16 dpp). Transcripts predominantly expressed at 0 and 4 dpp included homologs of histones, cyclins, and plastid and photosynthesis related genes. The group of genes with peak transcript levels at 8dpp included cytoskeleton, cell wall, lipid metabolism and phloem related proteins. This group was also dominated by genes with unknown function or without known homologs outside of cucurbits. A second shift in transcript profile was observed at 12-16dpp, which was characterized by abiotic and biotic stress related genes and significant enrichment for transcription factor gene homologs, including many associated with stress response and development.

**Conclusions:**

The transcriptome data coupled with morphological analyses provide an informative picture of early fruit development. Progressive waves of transcript abundance were associated with cell division, development of photosynthetic capacity, cell expansion and fruit growth, phloem activity, protection of the fruit surface, and finally transition away from fruit growth toward a stage of enhanced stress responses. These results suggest that the interval between expansive growth and ripening includes further developmental differentiation with an emphasis on defense. The increased transcript levels of cucurbit-specific genes during the exponential growth stage may indicate unique factors contributing to rapid growth in cucurbits.

## Background

Fleshy fruits are highly prized for nutritional content, flavor, fragrance, and appearance. While most fruits are eaten when ripe, a subset, including many that for culinary purposes are viewed as vegetables, are consumed immature. Cucumbers (*Cucumis sativus*), which are used as fresh product and processed into pickles, are typically harvested at the middle or end of the exponential growth phase, 1–2 weeks post-pollination, and approximately 2–3 weeks prior to fruit maturation.

Early fruit development is typified by phases of cell division and expansion [1]. In cucumber fruit, which develop from an enlarged inferior ovary, cell division occurs most rapidly prior to anthesis and then continues more slowly in the first 0–5 days post anthesis [2-5]. This phase largely overlaps with the period of highest respiration [4]. Fruit elongation begins almost immediately after pollination, with the most rapid increase occurring approximately 4–12 days post pollination (dpp) [6]. The rapid increase in cell size mirrors the rapid increase in fruit length, with obvious increase in vacuolization of mesocarp cells, and thickening in epidermal cell walls occurring between 8 and 16 dpp [6]. Cell division and expansion are largely completed by 12–16 dpp, with some variation depending on cultivar and season [4,6,7].

In addition to cell division and expansion, early development also includes specialized tissue and organ development and interaction with the abiotic and biotic environment. For example, developing cucumber fruit exhibit a distinct change in susceptibility to the soil-borne, oomycete pathogen, *Phytophthora capsici*; young fruit are highly susceptible, while older fruit are resistant [8,9]. There is a sharp transition in susceptibility that occurs at approximately 10–12 dpp coinciding with the end of the period of rapid fruit elongation. This age-related resistance suggests additional kinds of developmental changes occurring in the young cucumber fruit.

Although a limited number of studies have examined gene expression during early fruit development, a picture reflecting cell division and expansion is beginning to emerge based on transcriptomic studies of apple, cucumber, grape, tomato and watermelon. Among the enriched categories associated with tomato fruit set, were genes associated with protein biosynthesis, histones, nucleosome and chromosome assembly and cell cycle, suggesting a profile reflective of active cell division [10-12]. In contrast, various water, sugar and organic acid transport-associated genes were under-represented, but then increased with the transition from cell division to cell expansion. Highly expressed categories of genes expressed in expanding cucumber, as well as apple, grape, tomato, melon and watermelon fruits, included cytoskeleton and cell wall modifying genes such as tubulins, expansins, endo-1,2-B-glucanase, beta glucosidases, pectate lyases, and pectin methylesterases, and transport associated genes such as aquaporins, vacuolar H+ATPases, and phloem-associated proteins [6,10,13-18]. The most highly represented transcripts in rapidly expanding cucumber fruit (8 dpp) also were strongly enriched for defense related homologs including, lipid, latex, and defense-related genes, e.g., chitinase, thionin, hevein, snakin, peroxidase, catalase, thioredoxin, and dehydrins [6].

The early stages of fruit development, including fruit set and exponential growth, are clearly essential for all fruits. However, despite their importance as determinants of fruit size and yield, there has been little detailed analysis of this phase of development. Most studies to date, including recent transcriptomic studies, have focused on late development, or a broad range of developmental stages, with only a single snapshot during early development eg., [19-22]. In this study we combined morphological characterization with transcriptome analysis to provide new insight into important early fruit developmental stages and processes. Our observations, performed at five time points during the period from fruit set through the end of exponential fruit growth, indicate that this is a dynamic period of cucumber fruit development involving an array of internal and external morphological, physiological, and transcriptomic changes that act in concert with phases of active cell division, expansion, and response to the environment. Relative to anthesis and early fruit set, the period of peak- and late-exponential growth includes a large portion of highly represented transcripts, either of unknown function, or without homologs in Arabidopsis, suggesting unique factors contributing to the rapid growth phase in cucurbits. The end of exponential growth was marked by a shift in transcriptome profile characterized by abiotic and biotic stress related genes and significant enrichment for transcription factor gene homologs associated with stress response and development, suggesting that the interval between expansive growth and ripening may include a programmed transition toward enhanced defense.

## Results and discussion

### Morphological changes during early cucumber fruit development

**Figure 1:**
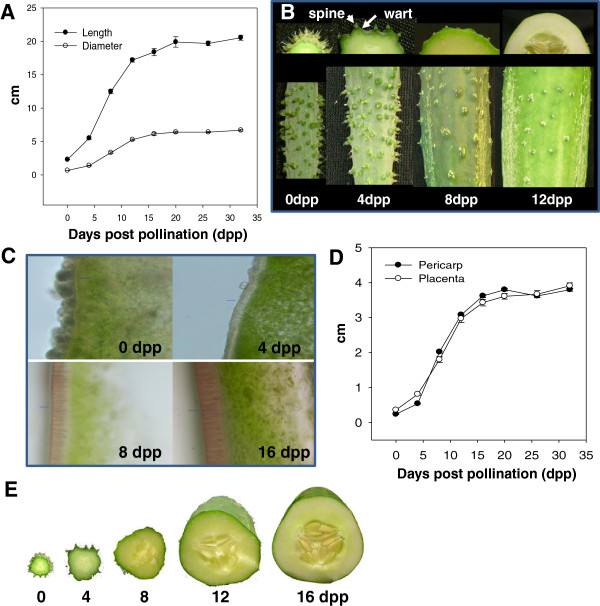
**Growth and development of cucumber fruits.** (**A**) Increase in fruit length and diameter as a function of days post pollination (dpp). (**B**) Changes in fruit surface during fruit growth including spine maturation and abscission, development and subsidence of warts, presence of ‘bloom’ and loss of chlorophyll. (**C**) Micrographs showing changes in fruit surface with age (magnification 200x; staining was with Sudan IV). (**D**) Thickness of fruit pericarp and placenta. (**E**) Cross section of developing cucumber fruit at 0, 4, 8, 12 and 16 dpp

Young Vlaspik cucumber fruit followed a highly reproducible progression of growth and development including visible external and internal morphological changes. Increase in size occurred rapidly after fertilization with most rapid growth occurring between 4 and 12 dpp (Figure 1A). After approximately 16 dpp, fruit size remained largely constant until fruit maturation at approximately 30 dpp. At 0 dpp (anthesis), deep ridges along the length of fruit covered the surface of the fruit. Densely spaced spines were randomly scattered relative to the ridges (Figure 1B). In contrast to ridges, which were most prominent at anthesis, warts, which are typically are formed at the base of spines, were diminutive at 0 dpp. They rapidly developed to become highly prominent at 4 dpp but then flattened out with further fruit expansion. Both ridges and warts were nearly absent by 12 dpp. The spines followed a maturation process culminating in abscission. At 0 dpp spine color was translucent light green. At approximately 8 dpp they started to senesce, turning yellow, then white at 12–16 dpp. By 16 dpp many had abscised from the fruit surface.

At anthesis, the exocarp was dark green. Dark green/light green stripes and specks on the surface of the fruit began to emerge around 8 dpp. The fruit surface at anthesis also has a dull appearance due to ‘bloom’ (Figure 1B), a fine white powder primarily composed of silica oxide (SiO2) [23]. The bloom disappeared first from the peduncle end around 4 dpp, then the blossom end by 8 dpp; by 12 dpp, it had disappeared completely, leaving a shiny fruit surface. The cuticle layer showed increased thickness with age. After 12 and 16 dpp it stained more darkly with Sudan IV, indicating increased cutin or wax content that appeared to penetrate between the pallisade cells in the epidermal layer (Figure 1C).

With respect to internal fruit morphology, both placenta and pericarp rapidly expanded from 4–16 dpp. The rate and amount of expansion was very similar for both tissues (Figure 1D). The mesocarp was initially green at 0 and 4 dpp, but became progressively lighter with age. Increase in mesocarp cell size is accompanied bv increased vacuolization between 4 and 12 dpp [6]. The placenta tissue became gelatinous between 8 and 12 dpp and hardening of seed coats occurred between 12 and 16 dpp (Figure 1E).

### 454 pyrosequencing data

454 pyrosequencing analysis of cDNA libraries prepared from pericarp RNA samples of fruit harvested 0, 4, 8, 12, and 16 days post-pollination provided 1.13 million reads (Additional file 1: Table S1). The resulting data were assembled into 27,859 contigs with a mean length of 834 base pairs (bp). All transcripts were mapped to the assembled cucumber genome of Huang et al. [24], although in some cases more than one transcript mapped to the same location. The number of the reads per contig ranged from 2 to more than 14,000 with a mean of 67 reads per contig and median of 7 reads/contig. Assembed contig length increased steadily with the number of ESTs/contig, until approximately 30 reads/contig where it leveled off with an average length of approximately 1400 bp (Additional file 2: Figure S1). Similarly, frequency of identification of homologs in *Arabidopsis* increased with number of ESTs/contig, leveling off at approximately 90% with approximately 30 reads/contig (Additional file 2: Figure S1).

Gene ontology (GO) assignment to those contigs with putative homologs in Arabidopsis showed a similar distribution of gene functions as are present in the full Arabidopsis genome (generally within 2-fold relative to the distribution in Arabidopsis), suggesting broad representation of the genome (Additional file 2: Figure S1). Approximately half of the contigs with ≥30 reads but without homologs in Arabidopsis had putative homologs in other species. The final portion, approximately 5% of the total (275 contigs), either did not have any identified homologs in the current NCBI nr database, or only had putative homologs in cucurbit species, suggesting that these transcripts may be unique to cucumber or cucurbits relative to the plant species sequenced to date. These potentially cucurbit-unique transcripts included 91 very highly expressed contigs, represented by at least 100 ESTs (average length >1000 bp). Eighteen had putative functional assignments, eight of which were known cucurbit specific phloem-related proteins, such as phloem lectins and phloem proteins (Table 1).

**Table 1:** Functional annotation of transcripts represented by greater than 30 ESTs with homologs only identified in cucurbit species

**No. reads**	**Contig #**	**Length (bp)**	**Best BLASTX hit**		**E value**
1,222	2152	815	gi|22023939	26 kDa phloem protein [*Cucumis sativus*]	2.0E-89
886	3111	1,010	gi|1753099	phloem filament protein; PP1; phloem protein 1 [*Cucurbita maxima*]	1.0E-25
546	2219	882	gi|21952270	26 kDa phloem lectin [*Cucumis sativus*]	4.0E-83
438	1685	625	gi|1669529	CRG16 (gibberelin responsive) [*Cucumis sativus*]	4.0E-27
315	372	825	gi|21745319	17 kDa phloem lectin [*Cucumis sativus*]	4.0E-62
312	1342	678	gi|21952272	phloem lectin [*Cucurbita argyrosperma subsp. sororia*]	8.0E-42
257	1071	857	gi|33415266	poly(A)-binding protein C-terminal interacting protein 6 [*Cucumis sativus*]	1.0E-72
139	4582	965	gi|21686470	26 kDa phloem lectin [*Cucumis melo*]	1.0E-55
130	2648	1,894	gi|219567000	galactose-binding type-2 ribosome-inactivating protein [*Momordica charantia*]	1.0E-130
129	2280	668	gi|94450551	pathogen induced 4 protein [*Cucumis sativus*]	9.0E-32
112	5677	1,666	gi|148270942	expressed protein [*Cucumis melo*]	1.0E-174
77	6868	575	gi|2406582	pathogen-induced protein CuPi1 [*Cucumis sativus*]	4.0E-46
62	6937	620	gi|2576407	seed nucellus-specific protein [*Citrullus lanatus*]	2.0E-40
54	3108	803	gi|21745319	17 kDa phloem lectin [*Cucumis sativus*]	5.0E-88
51	3170	696	gi|169219257	putative Gly-rich RNA-binding protein [*Cucumis sativus*]	4.0E-49
48	1586	901	gi|21686470	26 kDa phloem lectin [*Cucumis melo*]	4.0E-31
42	2896	563	gi|51537955	beta-caryophyllene synthase [*Cucumis sativus*]	2.0E-52
37	2099	733	gi|58263793	profilin [*Cucumis melo*]	1.0E-29
33	5950	1,288	gi|28558780	gag-protease polyprotein [*Cucumis melo*]	1.0E-80

### Changes in transcript abundance during early fruit growth

**Figure 2:**
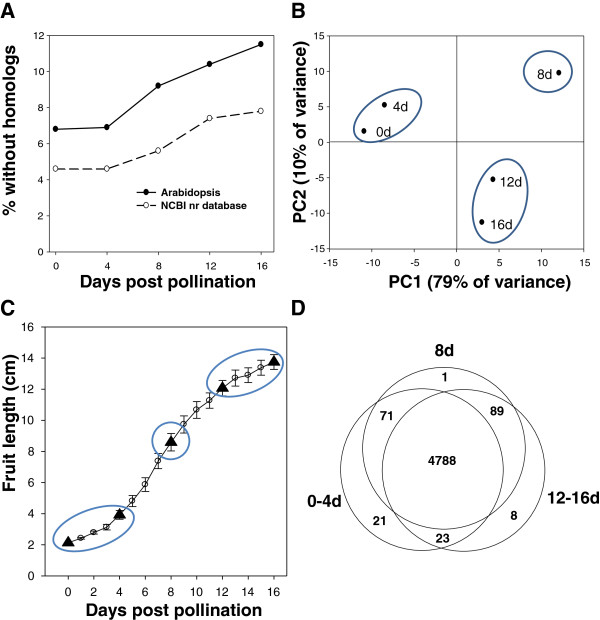
**Comparison of transcripts expressed in the cucumber fruit libraries.** (**A**) Portion of contigs represented by at least 30 ESTs in a given fruit age library [0, 4, 8, 12, or 16 days post pollination (dpp)] that did not have putative homologs in Arabidopsis or other sequences present in the NCBI nr database. (**B**) Principal component analysis of transcripts expressed at the five different fruit ages. (**C**) Relationship between ages grouped by principal component analysis and fruit growth. (**D**) Venn diagrams showing genes commonly expressed among the three age groups

Based on the observed relationship between ESTs/contig, contig length, and putative homologs in Arabidopsis (Additional file 2: Figure S1), subsequent bioinformatic analyses were performed on contigs represented by at least 30 ESTs. The distribution of contigs represented by at least 30 ESTs that did not have putative homologs outside of cucurbit species was not evenly distributed across fruit age (Figure 2A). The 8, 12, and 16 dpp libraries contained nearly twice as many contigs without identified homologs in Arabidopsis as was observed for the 0 and 4 dpp libraries. Of the 91 very highly abundant transcripts without known homologs outside of cucurbits, only three were not observed in the 8, 12 or 16 dpp samples. In contrast, 17 of the cucurbit specific transcripts did not appear in 0 or 4 dpp samples.

To validate usefulness of the 454 sequence data for analysis of transcript abundance, a set of fourteen genes representing different levels of EST representation/contig across the different fruit ages were selected for quantitative real time (qRT)-PCR analysis (Additional file 3 Figure S2). These included genes such as cyclin-dependent kinase B2;2 with high transcript levels early in development (0–4 dpp) or expansin A5 with higher transcript levels at 8–16 dpp. Comparison of transcript level at a given age relative to baseline expression at 0dpp (56 gene/time comparisons) showed good correspondence between values obtained by 454 sequencing and qRT-PCR (Pearson’s correlation, R^2^ = 0.85; Additional file 3: Figure S2). There was also good correspondence between the qRT-PCR results obtained from two different growth experiments in the greenhouse (R^2^ = 0.91), indicating biological reproducibility of patterns of gene expression across fruit ages, and validity of the use of frequency of EST representation in the 454 library as a measure of level of gene expression.

Principal component analysis (PCA) was performed on transcript levels among the libraries from the five fruit ages (Figure 2B). The first two components, which accounted for nearly 90% of the variation, separated the fruit ages into three groups, 0 and 4 dpp, 8 dpp, and 12 and 16 dpp. Examination of fruit growth rate indicated that these age groups correspond with cell division/pre-exponential growth, peak exponential expansion, and late/post-exponential expansion stages of growth, respectively (Figure 2C). Comparison of the transcripts present in each of the age groups showed that the great majority were detected in all three age groups. The fewest unique transcripts were present in the 8 dpp sample, consistent with a developmental gradient of transcription moving from 0–4 to 8 to 12–16 dpp. Both the PCA and Venn Diagram (Figure 2B,D) show the least commonality between the 0–4 and 12–16 dpp age groups.

The most highly represented contigs in each age group (0.1% of transcript pool; 79, 111 and 107 contigs for 0–4, 8, and 12-16dpp, respectively) exhibited markedly different profiles of putative gene function. Among those in common to all three groups were housekeeping genes including numerous ribosomal protein genes, and several tubulins, actins, and redox-related genes (catalase, ascorbate oxidase, ascorbate peroxidase), as well as several with unknown function or no identifiable homolog in Arabidopsis. Examination of the transcripts that were very highly represented in only one age group (Table 2), showed that 0–4 dpp was the only one to include histone genes. This observation is consistent with the high level of DNA replication occurring in young, dividing cells. Similarly, ribosomal protein genes were among the most highly represented transcripts at 0–4 dpp (8/21 genes) but minimally present in the 8 or 12–16 dpp ages (2 and 0 times, respectively). The 12–16 dpp group was marked by numerous abiotic stress related genes.

**Table 2:** Cucumber fruit contigs very highly expressed in only one age group (>0.1% representation) at 0–4, 8, or 12–16 dpp

**Contig #**	**Length** **(bp)**	**Reads**		**Hit ID Arabidopsis**	**Hit Description**	**E value**
		Total	0+4			
3017	2294	1346	1269	AT1G08450	CRT3 (CALRETICULIN 3); calcium ion binding/unfolded protein binding	0.0
2794	1211	1482	1100	AT4G35100	PIP3 (PLASMA MEMBRANE INTRINSIC PROTEIN 3); water channel	1.0E-139
1157	739	1256	792	AT1G26880	60S ribosomal protein L34 (RPL34A)	2.0E-59
2093	1884	1133	721	AT5G62700	TUB3; GTP binding/GTPase/structural molecule	0.0
2075	637	1185	657	AT2G21580	40S ribosomal protein S25 (RPS25B)	3.0E-51
886	1337	1195	633	AT3G55440	TPI (TRIOSEPHOSPHATE ISOMERASE); triose-phosphate isomerase	1.0E-119
2327	1622	858	615	AT4G27440	PORB (PROTOCHLOROPHYLLIDE OXIDOREDUCTASE B	0.0
3154	1085	1136	611	AT1G67430	60S ribosomal protein L17 (RPL17B)	1.0E-86
885	1370	951	605	AT2G30620	histone H1.2	1.0E-66
2351	608	1093	579	AT3G61110	ARS27A (ARABIDOPSIS RIBOSOMAL PROTEIN S27)	7.0E-46
2804	1198	1072	575	AT3G04840	40S ribosomal protein S3A (RPS3aA)	1.0E-126
2255	888	1075	566	AT4G40030	histone H3.2	4.0E-72
192	1383	701	522	AT1G18250	ATLP-1; thaumatin-like protein	1.0E-115
2894	744	641	518	AT5G59970	histone H4	2.0E-53
2685	1054	788	497	AT5G39850	40S ribosomal protein S9 (RPS9C)	1.0E-102
606	1477	756	492	***No hits found***		
1038	1121	948	484	AT3G53740	60S ribosomal protein L36 (RPL36B)	8.0E-45
820	702	783	481	AT5G50460	protein transport protein SEC61 gamma subunit, putative	9.0E-32
107	714	670	474	AT5G59970	histone H4	1.0E-53
1260	801	867	463	AT5G56710	60S ribosomal protein L31 (RPL31C)	3.0E-54
5501	1223	912	459	AT3G52590	UBQ1 (UBIQUITIN EXTENSION PROTEIN 1)	1.0E-68
		Total	8			
3064	960	1162	776	AT5G22430	***unknown protein***	1.0E-25
3334	1146	832	505	AT3G46040	RPS15AD (ribosomal protein S15A D)	2.0E-27
1282	680	980	490	***No hits found***		
1650	1644	514	404	AT5G33370	GDSL-motif lipase/hydrolase family protein	1.0E-146
3111	1010	886	349	***No hits found***	***phloem filament protein; PP1;phloem protein 1 [Cucurbitamaxima]***	1.0E-25
424	912	1035	331	AT5G59880	ADF3 (ACTIN DEPOLYMERIZING FACTOR 3); actin binding	5.0E-63
153	715	766	317	***No hits found***		
12937	953	418	312	AT4G15630	integral membrane family protein	3.0E-37
11169	949	792	284	AT1G28330	DYL1 (DORMANCY-ASSOCIATED PROTEIN-LIKE 1)	3.0E-41
2993	1046	494	270	***No hits found***		
2805	767	757	267	***No hits found***		
13496	951	687	267	***No hits found***		
2700	654	428	263	AT5G38650	proteasome maturation factor UMP1 family protein	2.0E-61
2357	598	526	261	***No hits found***		
1468	899	821	244	AT1G51200	zinc finger (AN1-like) family protein	8.0E-53
2350	973	551	242	AT1G11530	ATCXXS1; protein disulfide isomerase	2.0E-37
3297	1632	729	242	AT3G26960	***unknown protein***	8.0E-35
3067	1145	744	240	AT2G10940	protease inhibitor/seed storage/lipid transfer protein (LTP) family protein	5.0E-61
342	928	717	225	AT3G26960	***unknown protein***	2.0E-43
456	830	645	223	***No hits found***		
485	1612	919	220	AT1G09690	60S ribosomal protein L21 (RPL21C)	1.0E-81
924	1025	673	214	AT4G37300	MEE59 (maternal effect embryo arrest 59)	5.0E-24
1445	1372	514	203	AT5G65020	ANNAT2 (Annexin Arabidopsis 2); calcium ion binding	1.0E-131
620	1165	472	199	AT3G10210	***unknown function***	1.0E-94
2771	1339	491	195	***No hits found***		
489	829	799	192	AT1G79040	PSBR (photosystem II subunit R)	4.0E-56
3147	950	593	192	AT4G14305	***unknown function***	1.0E-62
		Total	12+16			
1561	899	1684	1221	***No hits found***		
504	1593	1167	855	AT5G47120	ATBI1 (BAX INHIBITOR 1)	1.0E-109
2496	1641	1319	810	AT2G02760	ATUBC2 (UBIQUITING-CONJUGATING ENZYME 2)	9.0E-86
2811	1259	1273	694	AT1G07890	APX1 (ascorbate peroxidase 1); L-ascorbate peroxidase	1.0E-119
493	3235	1307	679	***No hits found***	hypothetical protein [Vitis vinifera]	8.0E-37
1702	874	685	607	AT5G59720	HSP18.2 (heat shock protein 18.2)	2.0E-69
2864	1409	870	578	AT2G43750	OASB (O-ACETYLSERINE (THIOL) LYASE B); cysteine synthase	1.0E-142
1551	2577	765	558	AT5G19150	carbohydrate kinase family	1.0E-135
54	1237	1084	538	AT2G23090	***unknown protein***	4.0E-35
410	1047	1055	518	AT1G13950	ELF5A-1 (EUKARYOTIC ELONGATION FACTOR 5A-1)	6.0E-79
296	1842	750	516	AT3G44110	ATJ3; heat shock protein DNAJ homolog, protein binding	0.0
281	1330	1094	532	AT5G20720	CPN20 (CHAPERONIN 20); calmodulin binding	6.0E-98
2649	1071	1017	502	AT2G21660	CCR2 (COLD, CIRCADIAN RHYTHM, AND RNA BINDING 2)	7.0E-44
2541	2197	740	483	AT3G48000	ALDH2B4; 3-chloroallyl aldehyde dehydrogenase	0.0
698	1330	950	477	AT5G22080	DNAJ heat shock N-terminal domain-containing protein	1.0E-112
1568	1292	515	470	AT5G12020	HSP17.6II (17.6 KDA CLASS II HEAT SHOCK PROTEIN)	7.0E-49
1606	1153	687	455	AT2G43750	OASB (O-ACETYLSERINE (THIOL) LYASE B); cysteine synthase	1.0E-121
2828	1099	640	435	AT1G51200	zinc finger (AN1-like) family protein	4.0E-51
2414	869	613	432	AT3G16640	TCTP (TRANSLATIONALLY CONTROLLED TUMOR PROTEIN)	2.0E-70
2054	595	551	427	***No hits found***		
2219	882	546	411	***No hits found***	26 kDa phloem lectin [Cucumis sativus]	4.0E-83

Strikingly, genes with unknown function or without Arabidopsis homologs, dominated the group at 8 dpp, accounting for more than half of the contigs (14/27 genes, 52%).

The exponential growth stage of tomato also was associated with a larger proportion of ESTs with unknown function relative to other ages [10]. Fewer genes with unknown function or without Arabidopsis homologs occurred in the 12–16 dpp group (5/21) and only 1 member of the 0-4dpp group had no assigned putative function or was without a homolog in Arabidopsis.

**Figure 3:**
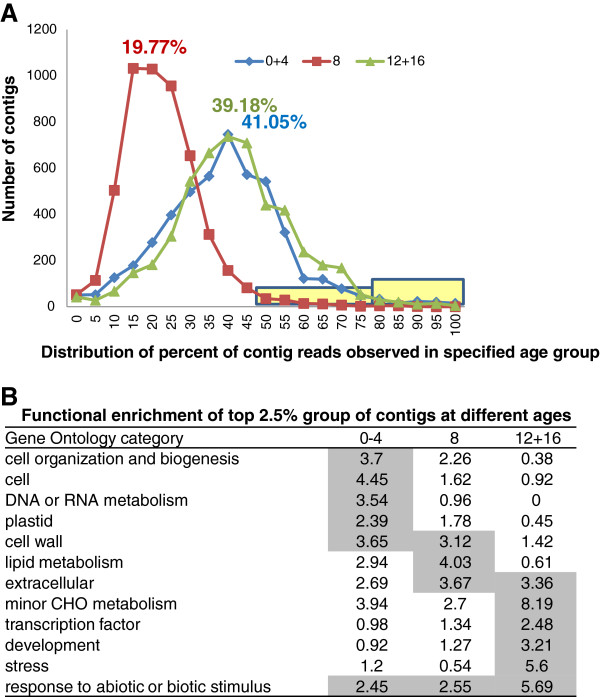
**Portion of gene expression observed in each age group and biological enrichment analysis.** (**A**) Distribution of the portion of gene expression observed at each age group for all contigs with ≥30 reads. Shading represents those contigs most strongly expressed for each of the age groups (top 2.5%). (**B**) Biological enrichment analysis of contigs with age group-enriched expression as identified in (A). Functional distribution, normalized frequency, and bootstrap standard deviation (SD) of contigs with putative Arabidopsis homologs was determined using the categories classification from the Classification SuperViewer from Bio-Array Resource for Arabidopsis Functional Genomics for Gene Ontology [25]. Shading indicates those categories that are significantly enriched (P < 0.05)

To identify less highly represented genes that were strongly enriched at a specific age group, contigs were normalized for portion of reads observed at different time points. If transcription levels were constant during development, 20% of the transcript reads would be observed at each of the five sample ages (i.e., 40% for 0+4dpp, 20% for 8dpp and 40% for 12+16dpp). Overall distribution of portion of transcripts observed at a given age followed this expectation for the transcriptome set, with a mean value of 41.05%, 19.77%, and 39.18%, respectively for 0+4, 8, and 12+16dpp age groups (Figure 3A). The tails of the distribution (top 2.5%) were examined for genes for which transcript levels were strongly enriched in a specific age group (approximately120 genes/group). This resulted in three non-overlapping sets of genes (Additional file 4: Table S2). There also was minimal overlap with the genes listed in Table 2. The genes listed in Table 2 had an average of 862 ESTs/contig whereas the mean number of ESTs/contig for the genes identified in this manner was 166. As was seen for the most highly represented group of genes, there was uneven distribution of genes without homologs in Arabidopsis or with unknown function; those accounted for 18.3% for 0+4 dpp enriched transcripts, but for 34.1% and 33.8% for 8dpp and 12+16 dpp enriched transcripts, respectively.

### Fruit set/pre-exponential growth

**Figure 4:**
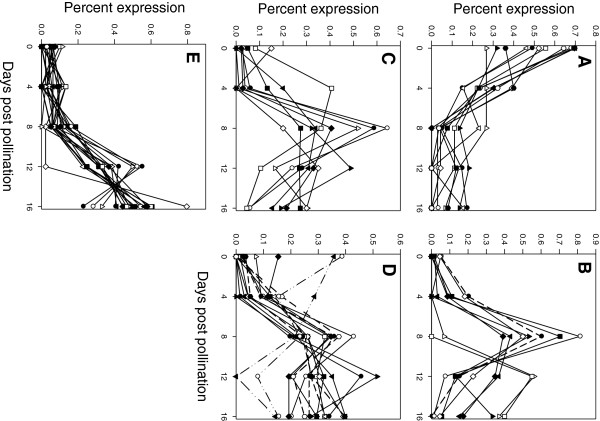
**Functional groups of genes showing age-specific expression.** (**A**) Expression of cyclin related genes relative to fruit age plotted as percent total expression for that transcript observed at each age [putative homologs of cyclins B1;2 (At5g06150), B1;4 (At2g26760); D1;1 (At1g70210);, D3;1 (At4g34160), D3;3 (At3g50070), D5;1 (At4g37630), and cyclin dependent kinases (CDK), CDKB1;2 (At2g38620), CDKB2;2 (At1g20930), CDKD1;3 (At1g18040), CDKE;1 (At5g63610); CKS1 (At2g27960)]. (**B**) GDSL-motif lipase/hydrolase family protein genes (putative homologs of At1g09390, At1g56670, At2g04570, At2g42990, At3g16370, At3g48460, At5g03610, At5g14450, At5g33370, At5g62930) and transcription factor *SHINE1* (At 1 g15360; dotted line). (**C**,) Lipid transfer protein (LTP) family protein genes (putative homologs of At1g48750, At1g62510, At2g10940, At2g45180, At5g01870, At5g64080). (**D**) Phloem proteins. Solid lines indicate cucurbit specific phloem proteins as listed in Table 1. Dotted lines indicate putative homologs to Arabidopsis phloem proteins (ATPP) 2-A genes (two for ATPP2-A1, one for ATPP2-A9, and ATPP2-A13); dashed lines are putative homologs of ATPP2-B genes (ATPP-B10 and ATPP-B12). (**E**) Transcription factors showing preferential expression at 12 +16 dpp (putative homologs of At1g27730, At1g50640, At2g17040, At2g26150, At2g40140, 2 g46240, At3g15210, At3g15510, At3g16770, AT3g56400, At4g11660, At4g16780, At4g39250, At5g25560, At5g51190)

Functional enrichment analysis of those transcripts with age-group enriched transcript levels indicated that the 0–4 dpp age group had significantly increased representation of genes associated with cell organization and biogenesis, and DNA or RNA metabolism, that subsided with age (Figure 3B). In addition to histone genes, which were also among the most highly abundant transcripts for this age group (Table 2), numerous putative cell cycle genes, cyclin- and cyclin dependent kinase-related gene family members, exhibited greater than 90% of transcript reads at 0–4 dpp (Additional file 4: Table S2, Figure 4A). Extensive protein interaction and gene expression data from Arabidopsis have allowed for the development of a picture of the cyclin interactome, including characterization of complexes associated with different cell phases [26]. Cyclin related genes strongly enriched at 0–4 dpp in the cucumber fruit transcriptome, such as putative homologs of CDKB1;2, CDKB2;2, CYCB1:2; CYCD3;1, CYCD3;3, CYCD5;1, were among those associated with the mitosis and post-mitosis (M and G1) phases in the Arabidopsis interactome. Elevated expression of several of these genes was also observed during fruit set in pollinated vs. unpollinated apple and cucumber flowers [3,27]. In contrast, the homolog of CDKA;1 [TAIR:At3g48750], which was uniformly represented in the young cucumber fruit transcriptome, was associated with cyclin complexes throughout the Arabidopsis cell cycle. 

**Figure 5:**
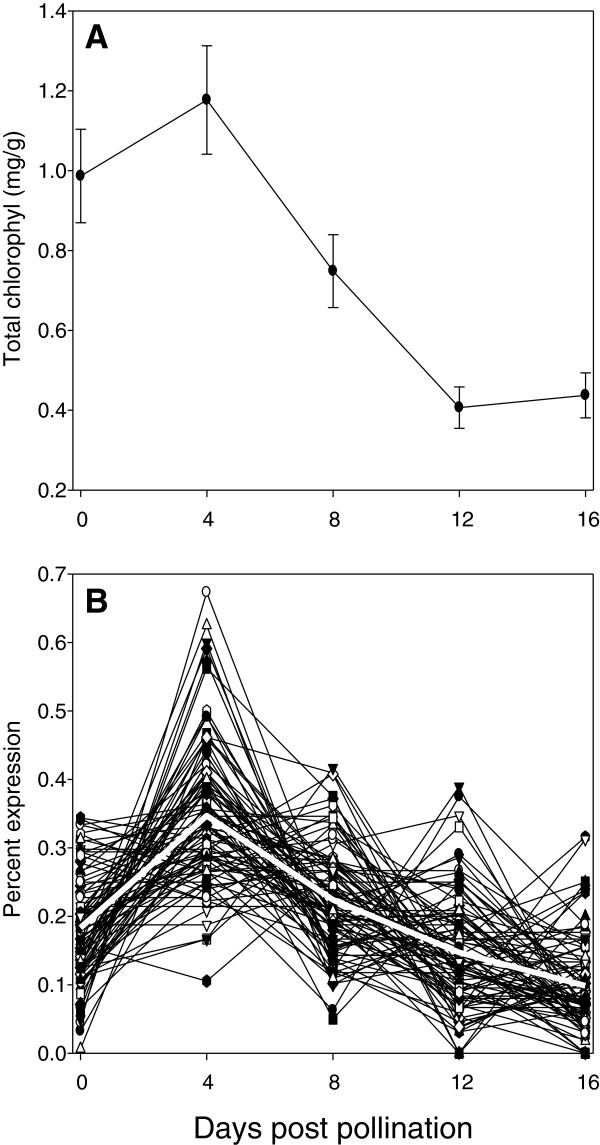
**Chlorophyll content and expression of chlorophyll and chloroplast-related transcripts in relationship to fruit age.** (**A**) Chlorophyll content. (**B**) Expression of chlorophyll and chloroplast-related transcripts in relationship to fruit age. Gene expression is plotted as percent of total expression observed for that transcript at each age. The heavy white line represents average percent gene expression at each age for the 91 genes (Supplemental file 2) with homologs in Arabidopsis annotated to be associated with chlorophyll or chloroplasts

The categories of plastid and chloroplast also were significantly enriched in the 0–4 dpp group, then declined with age. This is consistent with the decrease in chlorophyll observed after 4dpp; chlorophyll content per gram fresh weight peaked at 4 dpp, and then decreased until 12 dpp (Figure 5A). The assembled contigs included 91 transcripts whose homologs in Arabidopsis had annotations including one or more of the following terms: chlorophyll, chloroplast, photosystem, or thylakoid (Additional file 5: Table S3). Overall patterns of transcript abundance for these genes paralleled chlorophyll content in the developing fruit (Figure 5B).

**Figure 6:**
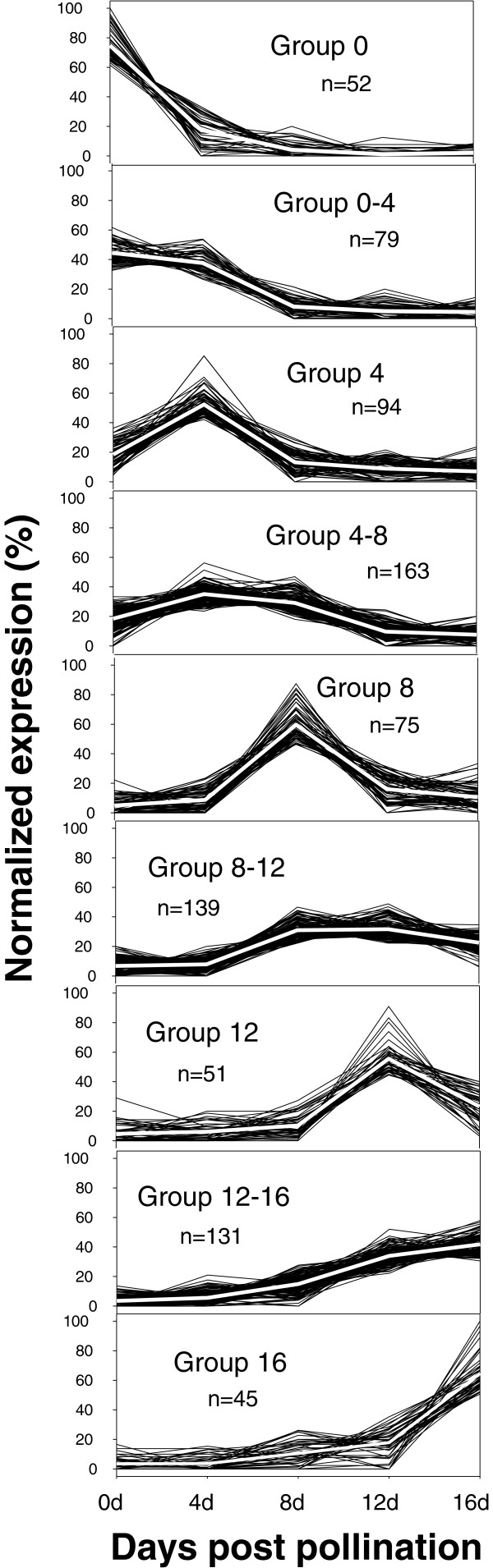
**Profiles of cucumber fruit transcripts showing age-specific expression patterns as determined by K-means analysis.** Analyses were performed using Cluster 3.0 software [25]

K-means cluster analysis allowed for further identification of transcripts showing progressive patterns of representation with fruit age (Figure 6). The chloroplast and other photosynthesis related genes described above, along with homologs of at least 10 additional chloroplast located proteins and enzymes predominated in the 4 or 4+8 clusters, but were minimally observed in the 0, 0+4, or 8 dpp clusters, and did not appear in the later clusters (Figure 6, Additional file 6: Table S4).

### Exponential growth

The group of genes with peak abundance at the 8 dpp, exponential growth stage, included cytoskeleton, cell wall, and water and carbohydrate transport genes. Tubulins, actin-related proteins, extensins, expansins, cellulose synthases, pectinase modifying enzymes, aquaporins, vacuolar H+ATPases, and phloem filament and lectin proteins, were among those strongly represented, as has been observed for other rapidly growing fleshy fruits such as tomato, apple, grape, and watermelon [10,13-15,17,18]. The major latex protein related genes also exhibited peak levels at 8 dpp, including two extremely highly transcribed genes that together accounted for more than 17,000 reads (Additional file 6: Table S4).

Putative homologs of vacuolar ATP synthase subunits B, D, H and P2 [TAIR:At4g38510, At3g58630, At3g42050, At1g19910] showed coordinate transcript abundance, with comparable levels increasing steadily until 8 dpp, and then gradually declining Two very highly represented homologs of the vacuolar aquaporin gene [TAIR:At2g36830], gamma tip tonoplast intrinsic protein, also peaked at 4-8dpp (Additional file 6 Table S4).

All of the cucurbit specific phloem proteins listed in Table 1 and the four putative homologs of the Arabidopsis phloem protein (ATPP) A2 family members observed in the data set peaked somewhat later, at 8–16 dpp with minimal transcript levels at 0 and 4 dpp (Figure 4D). Cucurbits are characterized by a unique and functionally divergent network of extrafascicular phloem external to the vascular bundles [28-30]. The highly expressed proteinaceous phloem filaments, comprised of the cucurbit-specific PP1 proteins, and the more widely distributed PP2 phloem lectin proteins [31], were found to be primarily associated with the extrafasicular phloem [30]. Strong expression of phloem protein genes during rapid growth has been observed in other studies, including PP1 expression in green stage watermelon fruit [18,31,32]. Specific expression of PP2 (a group A member [31]) was observed in young pumpkin (*Cucurbita pepo*) hypocotyls, peaking at 12 days after germination in concert with the period of peak growth and vascular differentiation [32]. In contrast, cucumber homologs of the ATPP2-B family had a nearly inverted pattern of transcript levels relative to PP2-A genes, peaking at 0 dpp, and dropping during exponential growth, suggesting possible functional divergence (Figure 4D).

The period of rapid fruit enlargement was also associated with marked changes in fruit surface, including an increase in cuticle thickness as is typically observed during rapid plant growth [33], and loss of the silica oxide powder based ‘bloom’. The homolog of the *Cucurbita moschata* silicon transporter [GenBank:327187680; ref. 23] showed age specific transcript abundance peaking at 8 dpp then dropping sharply, coinciding with the time of bloom loss from the middle of the fruit (the region from which samples were taken).

Among the genes identified in other systems to be associated with cuticle biosynthesis are the extracellular GDSL motif lipase/hydrolase proteins and lipid transfer proteins, which have been implicated in lipid transport to extracellular surfaces [33-36]. The cucumber fruit transcriptome set included eleven GDSL motif lipase/hydrolase protein family members that were represented by at least 30 ESTs, including five with more than 100 ESTs. The majority showed peak levels at 8 or 12–16 dpp, with virtually no measured reads until either 8 or 12 dpp (Figure 4B). Twelve lipid transfer protein (LTP) family members with greater than 30 ESTs/contig also were observed in the transcriptome data set, including four with greater than 700 ESTs. As for the GDSL motif lipase/hydrolase protein genes, the majority of the lipid transfer proteins were most highly represented from 8–16 dpp; transcript levels of one gene peaked at 4–8 dpp (Figure 4C).

A homolog of the transcription factor gene *SHINE1* [TAIR:At1g15360], which is associated with cuticle production in Arabidopsis (Figure 4B) [37] also exhibited peak transcript abundance at 8 dpp. Additionally, transcript levels of two cyctochrome P450 family members (CYP86A and CYP77A) that have been associated with cutin biosynthesis [38]; and two putative beta amyrin synthases, enzymes which have been associated with cuticular wax synthesis in tomato [39], also peaked at 8dpp (Additional file 4: Table S2). In contrast, two putative GDSL family members and one lipid transfer protein with moderate transcript levels (45–55 ESTs) [homologs of TAIR:At5g62930, At5g03610, and At2g45180, respectively] were observed almost exclusively at 0 dpp, suggesting possible floral, rather than fruit, expression (Additional file 6: Table S4).

### Late/post exponential growth

Stress-related genes (response to stress and response to abiotic and biotic stimulus categories) were over-represented at all stages, but considerably more so at 12–16 dpp than at the younger ages of 0–4 and 8 dpp (Figure 3B). The 12+16 dpp age group had the highest representation of abiotic and biotic stress related genes, including a variety of heat shock, redox, biotic defense and ethylene-related transcripts (Additional file 4: Table S2). Of the 120 genes in this group, 44 have high homology with genes associated with plant stress, including at least 13 transcription stress-related factors such as WRKY70 activator of SA-dependent defense; radical induced cell death; ethylene response, salt stress, and heat shock transcription factors (Figure 4E; Additional file 4: Table S2).

Overall, the group of genes with peak abundance at 12+16 dpp was significantly enriched for transcription factor genes (2.48-fold enrichment normalized frequency relative to Arabidopsis; P value = 3.19, E-04) (Figure 3B) accounting for 16% of the top 2.5% set. This may be contrasted with the total cucumber fruit transcriptome data set where transcription and transcription factor activity related genes were represented at a normalized frequency of 0.94 relative to occurrence in the Arabidopsis genome. Transcription factors in the top 2.5% of 0+4 and 8 dpp groups also were represented at a comparable frequency to the Arabidopsis genome, accounting for 3.7% and 4.6% of the gene list, respectively.

In addition to the stress related transcription factors with specific representation at 12–16 dpp, several putative transcription factor homologs were annotated to be associated with development [e.g., embryo sac development (*BEL1-LIKE HOMEODOMAIN* 1), morphogenesis (anac036/NAC domain containing protein 36), and cell expansion (ATHB-2 homeobox protein) (Additional file 4: Table S4). Furthermore, transcripts of other genes with homologs that have been implicated in development related processes are specifically observed at 12–16 days, such as putative homologs of *TCTP (TRANSLATIONALLY CONTROLLED TUMORPROTEIN*); *BTB AND TAZ DOMAINPROTEIN 1*; calcium-binding EF hand family protein; seed development related (*E12A11);* and *BAX INHIBITOR 1*.

## Conclusions

**Figure 7:**
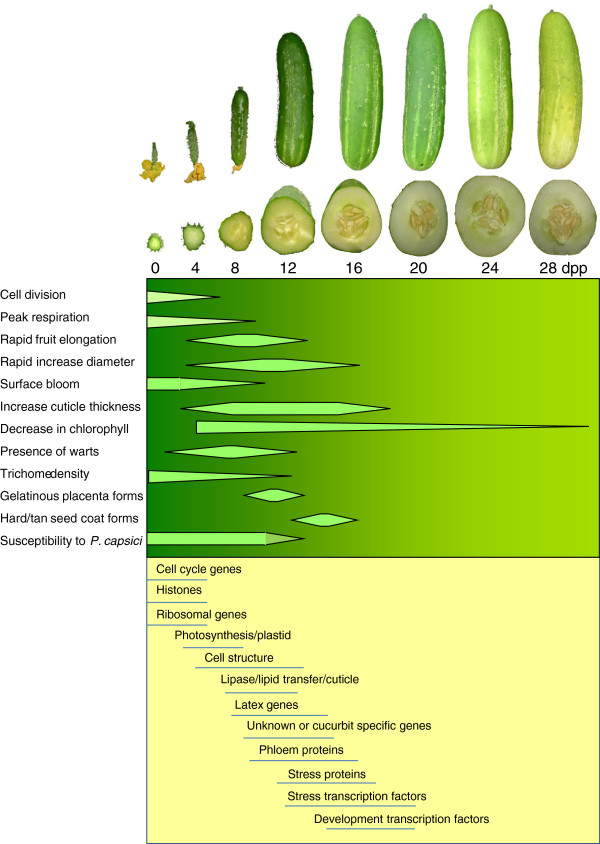
**Schematic representation of morphological and gene expression changes occurring during early cucumber fruit development.** Gene expression data refer to periods of peak expression for indicated gene categories. Data for respiration, cell division, and susceptibility to *P. capsici* are from Marcelis and Hofman-Eijer [4], Colle et al. [7], and Gevens et al. [9], respectively

Examination of early cucumber fruit growth from the period of pollination and initial fruit set through the end of the exponential growth phase shows a dynamic series of physiological and morphological changes (Figure 7). Transcriptomic analysis of the predominant genes represented in the different age groups as identified either by total number of reads (most highly represented among the genes at that age), portion of transcript reads observed at that age, or genes grouped by K-means cluster analysis, told a story aligned with the sequential stages of development.

Transcript representation in the youngest ages, 0–4 dpp, was uniquely characterized by genes associated with cell division, cell organization and biogenesis. At 4 dpp, transcription of the cell cycle genes was declining, while chloroplast, photosynthesis, and chloroplast-localized genes were peaking. Transcripts highly abundant during the exponential growth phase, 4–12 dpp, included extensive representation of genes associated with cell structure such as cytoskeleton, vacuoles and cell walls, along with surface lipid metabolism related genes, in concert with the period of greatest increase in cuticle thickness.

A second shift in the transcriptome profile was observed at 12–16 dpp with significant enrichment of abiotic and biotic stress related genes and stress-related and developmental transcription factor gene homologs. The enriched representation of numerous transcription factors relative to earlier ages suggests a programmatic change away from fruit growth, toward defense, and ultimately fruit maturation. This is also the time period where we have observed transition of cucumber fruit from susceptibility to resistance to *P. capsici*[8,9]. Classically, fleshy fruit development is described to consist of three stages post pollination: cell division, cell expansion, and ripening [1]. These results suggest that the interval between expansive growth and ripening may include further developmental differentiation; an emphasis on defense would be consistent with the role of fruit in protecting the developing seeds during embryo maturation prior to facilitating seed dispersal.

Finally, approximately 5% of the contigs represented by ≥30 reads either did not have identified putative homologs, or did not have homologs outside of cucurbits suggesting potentially unique genes specific to cucumber or cucurbits. The observation that these genes, as well as genes with homologs but with no annotated function, rarely occurred in the 0–4 dpp group, suggests commonality among processes associated with early fruit set and cell division and/or greater knowledge about the fruit set stage. The predominance of transcripts without non-cucurbit homologs or with unknown predicted functions during the peak exponential growth stage may reflect fewer studies to date about this phase of growth, or unique adaptations of cucurbits to allow for extreme fruit growth rates associated with these species.

Collectively, the transcriptomic information provided by the young cucumber fruit samples coupled with morphological analyses provide an informative picture of early fruit development characterized by phases of active cell division, fruit expansion including novel or uncharacterized genes, and response to the environment, as summarized in Figure 7. The progressive modules of transcript abundance tell a story of cell division, development of photosynthetic capacity, cell expansion and fruit growth, phloem activity, protection of the fruit surface, and finally transition away from fruit growth toward defense and maturation.

## Methods

### Plant material, fruit growth, chlorophyll and cuticle measurements

Sets of 80 cucumber plants per experiment (pickling type, cv. Vlaspik; Seminis Vegetable Seed Inc, Oxnard, CA) were grown in the greenhouse in 3.78 L plastic pots filled with BACCTO (Michigan Peat Co., Houston, TX) media and fertilized once per week. Temperature was kept between 21 to 25°C, supplemental lights were used to provide an 18 h light period. Pest control was performed according to standard management practices. All flowers for each experiment were hand pollinated on a single date (1–2 flowers per plant). The experiment was repeated three times. Prior to the harvests, which were performed at 4 day intervals from 0–16 dpp, fruit were measured for length and diameter, and examined for external appearances including: presence or absence of wax along the length of the fruit; wart development; color patterns (e.g., stripes); and changes in presence, color, and densities of spines. Pericarp and placenta size was measured from the cross section of the fruit after harvest.

Exocarp samples (upper 1 to 2 mm) for chlorophyll measurement were removed by fruit peeler from the center portion of five fruit at each age and stored at – 20°C. Samples were subsequently thawed at room temperature and blotted on paper to remove excess water and 1 g gram portions were immersed in *N, N*-dimethylformamide for at least 24 hours at 4°C in dark. Total chlorophyll was calculated based on spectrophotometer absorbance measurements at 665 and 647 nm [40]. Samples to measure cuticle thickness were stained with Sudan IV(as per [41]) and measured using a Spot RT3 Digital Camera System at 200x magnification (SPOT Imaging Solutions, Diagnostic Instruments, Inc., MI).

### cDNA library production and 454 sequencing

Randomly assigned groups of twenty fruit were harvested at 0, 4, 8, 12, and 16 dpp and ranked by size; the middle ten fruits were used for RNA extraction. Pericarp samples consisting of exocarp, mesocarp, and placenta tissue but not seeds, were isolated from the center portion of the fruit by razor blade, immediately frozen in liquid nitrogen, and stored at – 80°C until RNA was isolated. Samples from ten fruits were pooled for RNA extraction; RNA and oligo(dT)-primed cDNA sample preparation were based on the procedures of Schilmiller et al. [42] and Ando and Grumet [6]. Final concentration was assessed by the nanodrop ND-1000 method and subsequent steps for 454 Titanium (0, 4, 1,2, 16 dpp) pyrosequencing analysis were performed by the Michigan State University Research Technology Support Facility (RTSF). Each sample was loaded on a 1/4 plate 454 Pico TiterPlate (454 Life Sciences, a Roche Corporation, CT). The 8 dpp sample was sequenced previously [6].

### Contig assembly and gene annotation

Contigs were assembled by the MSU RTSF Bioinformatics Group. Reads were processed through The Institute for Genomic Research (TIGR) SeqClean pipeline to trim residual sequences from the cDNA preparation, poly(A) tails and other low quality or low complexity regions [43]. Trimmed sequences were assembled into contigs using the TIGR Gene Indices Clustering Tools (TIGCL) [44]. Stringent clustering and alignment parameters were used to limit the size of clusters for assembly. Contigs from the first pass of assembly were then combined and subjected to a second assembly pass with CAP3 [45]. Less stringent alignment parameters were used for this pass to allow for minor sequencing errors or allelic differences in the cDNA sequence. Read data for 8 day post pollination samples is available from the Sequence Read Archive (SRA), accessible through NCBI BioProject ID PRJNA79541. Read data for 0, 4, 12 and 16 dpp samples in SRA as well as assembled contig sequences deposited as Transcriptome Shotgun Assemblies (TSA) and expression profiling data in the Gene Expression Omnibus (GEO) are available through NCBI BioProject ID PRJNA169904.

To estimate relative expression, the number of reads originating from each cDNA library were counted for each contig and reported relative to the total number of reads generated for that library as transcripts per thousand (TPT). The final contigs were subjected to BLASTX search against the green plant subdivision of the NCBI nr protein database and/or the Arabidopsis protein (TAIR9) databases to search for similarity to previously identified genes and assign possible gene functions. BLASTN analysis was performed for highly expressed contigs for which homologs were not identified by BLASTX searches.

### Transcriptome analysis

The Classification SuperViewer Tool w/Bootstrap web database [25] was used for GO categorization, determination of normalized frequencies relative to Arabidopsis, and calculation of bootstrap standard deviations, and P-values. Princomp procedure SAS 9.1 (SAS Institute, Cary, NC) was used for principal component analysis. The first two principal components, which explain nearly 90% of the total variation were extracted from the covariance matrix. To examine relative gene expression at each age, the portion of reads for that transcript relative to total reads for the transcript, was calculated for each transcript with >30 reads, for each age. Expression profiles were clustered by K-means method using Cluster 3.0 software [46].

### qRT-PCR

Total RNA was isolated and assessed for quality and quantity as above. RT reactions were performed using the High Capacity RNA-to-cDNA kit (Applied Biosystems, Foster City, CA). Gene-specific primers (Additional file 7: Table S5) were designed using Primer Express software. ABI Prism 7900HT Sequence Detection System was used for qRT-PCR analysis. Power SYBR Green PCR Master Mix (Applied Biosystems) was used for PCR quantification. Actin from *C. sativus* was used as an endogenous control and for normalization. Each qRT experiment was repeated three times. PCR products from each gene were quantified with reference to corresponding standard curves.

## Abbreviations

Dpp: Days post pollination; EST: Expressed Sequence Tag; GO: Gene Ontology; PCA: Principal Component Analysis; qRT-PCR: quantitative Real Time PCR.

## Competing interests

The authors declare that they have no competing interests.

## Authors’ contributions

R.G. and K.A. designed the study, analyzed the data and wrote the manuscript. K.A. performed the experimental work. K.C. performed the contig assembly and gene annotation. All the authors read and approved the final manuscript.

## Supplementary Material

Additional file 1 **Table S1.** Summary of 454 sequencing results and contig assembly for cucumber fruit libraries 0–16 days post pollination. Click here for file

Additional file 2 **Figure S1.** Relationship between number of ESTs per contig, mean contig length, and percent of contigs with homologs in Arabidopsis. Click here for file

Additional file 3 **Figure S2.** qRT-PCR verification of gene expression changes. Click here for file

Additional file 4 **Table S2.** Genes differentially expressed at 0–4, 8, or 12–16 dpp (top 2.5% at each age group). Click here for file

Additional file 5 **Table S3.** Transcripts with homologs in Arabidopsis annotated to include one or more of the following terms: chlorophyll, chloroplast, photosystem, thylakoid. Click here for file

Additional file 6 **Table S4.** Gene list for K-means cluster analysis groups. Click here for file

Additional file 7 **Table S5.** Primers used for qRT-PCR analysis. Click here for file

## References

[B1] Gillaspy G Ben-David H Gruissem W Fruits: A developmental perspective Plant Cell 1993 5 1439 1451 12271039 10.1105/tpc.5.10.1439 PMC160374

[B2] Boonkorkaew P Hikosaka S Sugiyama N Effect of pollination on cell division, cell enlargement, and endogenous hormones in fruit development in a gynoecious cucumber Scientia Hortic 2008 116 1 7 10.1016/j.scienta.2007.10.027

[B3] Fu FQ Mao WH Shi K Zhou YH Asami T Yu JQ A role of brassinosteroids in early fruit development in cucumber J Exp Bot 2008 9 2299 2308 18515830 10.1093/jxb/ern093 PMC2423651

[B4] Marcelis LFM Hofman-Eijer LRB Cell division and expansion in the cucumber fruit J Hortic Sci 1993 68 665 671

[B5] Robinson RW Decker-Walters DS Cucurbits 1997 CAB International, New York, NY

[B6] Ando K Grumet R Transcriptional profiling of rapidly growing cucumber fruit by 454-pyrosequencing analysis J Amer Soc Hortic Sci 2010 135 291 302

[B7] Colle M Shaaban M Grumet R Characterization of component factors associated with differences in cucumber fruit size and shape HortSci 2011 46 S281

[B8] Ando K Hammar S Grumet R Age-related resistance of diverse cucurbit fruits to infection by Phytophthora capsici J Amer Soc Hortic Sci 2009 134 176 182

[B9] Gevens AJ Ando K Lamour KH Grumet R Hausbeck MK Development of a detached cucumber fruit assay to screen for resistance and effect of fruit age on susceptibility to infection by Phytophthora capsici Plant Dis 2006 90 1276 1282 10.1094/PD-90-1276 30780932

[B10] Lemaire-Chamley M Petit J Garcia V Just D Baldet P Germain V Fagard F Mouassite M Cheniclet C Rothan C Changes in transcriptional profiles are associated with early fruit tissue specialization in tomato Plant Physiol 2005 139 750 769 10.1104/pp.105.063719 16183847 PMC1255993

[B11] Pascual L Blanca JM Canizares J Nuez F Analysis of gene expression during the fruit set of tomato: a comparative approach Plant Sci 2007 173 609 620 10.1016/j.plantsci.2007.07.006

[B12] Wang H Schauer N Usadel B Frasse P Zouine M Hernould M Latche A Pech J-C Fernie AR Bouzayen M Regulatory features underlying pollination-dependent and -independent tomato fruit set revealed by transcript and primary metabolite profiling Plant Cell 2009 21 1428 1452 10.1105/tpc.108.060830 19435935 PMC2700536

[B13] Amemiya T Kanayama Y Yamaki S Yamada K Shiratake K Fruit-specific V-ATPase suppression in antisense-transgenic tomato reduces fruit growth and seed formation Planta 2006 223 1272 1280 10.1007/s00425-005-0176-x 16322982

[B14] Janssen BJ Thodey K Schaffer RJ Alba R Balakrishnan L Bishop R Bowen JH Crowhurst RN Gleave AP Ledger S McArtney S Pichler FB Snowden KC Ward S Global gene expression of apple fruit development from the floral bud to ripe fruit BMC Plant Biol 2008 8 16 10.1186/1471-2229-8-16 18279528 PMC2287172

[B15] Lee YP Yu GH Seo YS Han SE Choi YO Kim D Mok IG Kim WT Sung SK Microarray analysis of apple gene expression engaged in early fruit development Plant Cell Rep 2007 26 917 926 10.1007/s00299-007-0308-9 17294193

[B16] Mascarell-Creus A Canizares J Vilarrasa-Blasi J Mora-Garcia S Blanca J Gonzalez-Ibeas D Saladie M Roig C Deleu W Pico-Silvent B Lopez-Bigas N Aranda MA Garcia-Mas J Nuez F Puigdomenech P Cano-Delgado AI An oligo-based microarray offers novel transcriptomic approaches for the analysis of pathogen resistance and fruit quality traits in melon (Cucumis melo L.) BMC Genomics 2009 10 467 10.1186/1471-2164-10-467 19821986 PMC2767371

[B17] Schlosser J Olsson N Weis M Reid K Peng F Lund S Bowen P Cellular expansion and gene expression in the developing grape (Vitis vinifera L.) Protoplasma 2008 232 255 265 10.1007/s00709-008-0280-9 18421552

[B18] Wechter WP Levi A Harris KR Davis AR Fei Z Katzir N Giovannoni JJ Salman-Minkov A Hernandez A Thimmapuram J Tadmor Y Portnoy V Trebitsh T Gene expression in developing watermelon fruit BMC Genomics 2008 9 275 10.1186/1471-2164-9-275 18534026 PMC2440768

[B19] Guo SG Liu JG Zheng Y Huang MY Zhang HY Gong GY He HJ Ren Y Zhong SL Fei ZJ Xu Y Characterization of transcriptome dynamics during watermelon fruit development: sequencing, assembly, annotation and gene expression profiles BMC Genomics 2011 12 454 10.1186/1471-2164-12-454 21936920 PMC3197533

[B20] Manganaris GA Rasori A Bassi D Geuna F Ramina A Tonutti P Bonghi C Comparative transcript profiling of apricot (Prunus armeniaca L. fruit development and on-tree ripening Tree Genet Genom 2011 7 609 616 10.1007/s11295-010-0360-4

[B21] Portnoy V Diber A Pollock S Karchi H Lev S Tzuri G Harel-Beja R Forer R Portnoy VH Lewinsohn E Tadmor Y Burger J Schaffer A Katzir N Use of non-normalized, non-amplified cDNA for 454-based RNA sequencing of fleshy melon fruit Plant Genome 2011 4 36 46 10.3835/plantgenome2010.11.0026

[B22] Zenoni S Ferrarani A Giancomelli E Xumerle L Fasoli M Malerba G Bellin D Pezzotti M Delledonne M Characterization of transcriptional complexity during berry development in Vitis vinifera using RNA-Seq Plant Physiol 2010 152 1787 1795 10.1104/pp.109.149716 20118272 PMC2850006

[B23] Mitani N Yamaji N Ago Y Iwasaki K Ma JF Isolation and functional characterization of an influx silicon transporter in two pumpkin cultivars contrasting in silicon accumulation Plant J 2011 66 231 240 10.1111/j.1365-313X.2011.04483.x 21205032

[B24] Huang S Li R Zhang Z Li L Gu X Fan W Lucas WJ Wang X Xie B Ni P Ren Y Zhu H Li J Lin K Jin W Fei Z Li G Staub J Kilian A van der Vossen EAG Wu Y Guo J He J Jia J Ren Y Tan G Lu Y Ruan J Qian W Wang M Huang Q Li B Xuan Z Cao J San A Wu Z Ahang J Cai Q Bai Y Zho B Han Y Li Y Li X Wang S Shi Q Liu S Cho WK Kim JY Xu Y Heller-Uszynska K Miao H Cheng Z Zhang S Wu J Yang Y Kang H Li M Liang H Ren X Shi A Wen M Jian M Yang H Zhang G Yang Z Chen R Liu S Li J Ma L Liu H Zhou Y Zhao J Fang X Li G Fang L Li Y Liu D Zheng H Zhang Y Qin N Li Z Yang G Yang S Bolund L Kristiansen K Zheng H Li S Zhang X Yang H Wang J Sun R Zhang B Jiang S Wang J Du Y Li S The genome of the cucumber, Cucumis sativus L Nature Genet 2009 41 1275 1281 10.1038/ng.475 19881527

[B25] Provart N Zhu T A browser-based functional classification SuperViewer for Arabidopsis genomics Curr Compu Molec Biol 2003 2003 271 272

[B26] Van Leene J Hollunder J Eeckhout D Persiau G Van De Slijke E Stals H Van Isterdal G Verkest A Neirynck S Buffel Y De Bodt S Maere S Laukens K Pharazyn A Ferreira PCG Eloy N Renne C Meyer C Faure JD Steinbrenner J Beynon J Larkin JC Van de Peer Y Hilson P Kuiper M De Veylder L Van Onckelen H Inze D Witters E De Jaeger G Targeted interactomics reveals a complex core cell cycle machinery in Arabidopsis thaliana Molec Sys Biol 2010 6 37 Article 397 10.1038/msb.2010.53 PMC2950081 20706207

[B27] Malladi A Johnson LK Expression profiling of cell cycle genes reveals key facilitators of cell production during carpel development, fruit set, and fruit growth in apple (Malus x domestica Borkh.) J Exp Bot 2011 62 205 219 10.1093/jxb/erq258 20732881 PMC2993910

[B28] Clark AM Jacobsen KR Bostwick DE Dannenhoffer JM Skaggs MI Thompson GA Molecular characterization of a phloem specific- gene encoding the filament protein, phloem protein 1 (PP1), from Cucurbita maxima Plant J 1997 12 49 61 10.1046/j.1365-313X.1997.12010049.x 9263452

[B29] Turgeon R Wolf S Phloem transport: Cellular pathways and molecular trafficking Annu Rev Plant Biol 2009 60 207 221 10.1146/annurev.arplant.043008.092045 19025382

[B30] Zhang B Tolstikov V Turnbull C Hicks LM Fiehn O Divergent metabolome and proteome suggest functional independence of dual phloem transport systems in cucurbits Proc Nat Acad Sci USA 2010 107 13532 13537 10.1073/pnas.0910558107 20566864 PMC2922161

[B31] Dinant S Clark AM Zhu YM Vilaine F Palauque JC Kusiak C Thompson GA Diversity of the superfamily of phloem lectins (phloem protein 2) in angiosperms Plant Physiol 2003 131 114 128 10.1104/pp.013086 12529520 PMC166792

[B32] Dannenhoffer JM Schulz A Skaggs MI Bostwick DE Thompson GA Expression of the phloem lectin is developmentally linked to vascular differentiation in cucurbits Planta 1997 201 405 414 10.1007/s004250050083

[B33] Yeats TH Howe K Matas AJ Buda GJ Thannhauser TW Rose JKC Mining the surface proteome of tomato (Solanum lycopersicum) fruit for proteins associated with cuticle biogenesis J Exp Bot 2010 61 3759 3771 10.1093/jxb/erq194 20571035 PMC2921210

[B34] Mintz-Oron S Mandel T Rogachev I Feldber L Lotan O Yativ M Wang Z Jetter R Venger I Adato A Aharoni A Gene expression and metabolism in tomato fruit surface tissues Plant Physiol 2008 147 823 851 10.1104/pp.108.116004 18441227 PMC2409049

[B35] Samuels L Kunst L Jetter R Sealing plant surfaces: cuticular wax formation by epidermal cells Annu Rev Plant Biol 2008 59 683 707 10.1146/annurev.arplant.59.103006.093219 18251711

[B36] Suh MC Samuels AL Jetter R Kunst L Pollard M Ohlrogge J Beisson F Cuticular lipid composition, surface structure, and gene expression in Arabidopsis stem epidermis Plant Physiol 2005 139 1649 1665 10.1104/pp.105.070805 16299169 PMC1310549

[B37] Aharoni A Dixit S Jetter R Thoenes E van Arkel G Pereira A The SHINE clade of AP2 domain transcription factors activates wax biosynthesis, alters cuticle properties and confers drought tolerance when overexpressed in Arabidopsis Plant Cell 2004 16 2463 2480 10.1105/tpc.104.022897 15319479 PMC520946

[B38] Li-Beisson Y Pollard M Sauveplane V Pinot F Ohlrogge J Beisson F Nanoridges that characterize the surface morphology of flowers require the synthesis of cutin polyester Proc Nat Acad Sci USA 2009 51 22008 22013 19959665 10.1073/pnas.0909090106 PMC2788479

[B39] Wang ZH Guhling O Yao RN Li FL Yeats TH Rose JKC Jetter R Two oxidosqualene cyclases responsible for biosynthesis of tomato fruit cuticular triterpenoids Plant Physiol 2011 155 540 552 10.1104/pp.110.162883 21059824 PMC3075788

[B40] Inskeep WP Bloom PR Extinction coefficients of chlorophyll a and b in N, N-dimethylformanide and 80% acetone Plant Physiol 1985 77 483 485 10.1104/pp.77.2.483 16664080 PMC1064541

[B41] Buda GJ Isaacson T Matas AJ Paolillo DJ Rose JK Three-dimensional imaging of plant cuticle architecture using confocal scanning laser microscopy Plant J 2009 60 378 385 10.1111/j.1365-313X.2009.03960.x 19563439

[B42] Schilmiller AL Schauvinhold I Larson M Xu M Charbonneaua AL Schmidt A Wilkerson C Last RL Pichersky E Monoterpenes in the glandular trichomes of tomato are synthesized from a neryl diphosphate precursor rather than geranyl diphosphate Proc Nat Acad Sci USA 2009 106 0865 10870 10.1073/pnas.0904113106 PMC2705607 19487664

[B43] The Institute for Genomic Research (TIGR) http://compbio.dfci.harvard.edu/tgi/software

[B44] Pertea G Huang X Liang F Antonescu V Sultana R Karamycheva S Lee Y White J Cheung F Parvizi B Tsai J Quackenbush J TIGR gene indices clustering tools (TIGCL): a software system for fast clustering of large EST databases Bioinformatics 2003 19 651 652 10.1093/bioinformatics/btg034 12651724

[B45] Huang X Madan A CAP3: A DNA sequence assembly program Genome Res 1999 9 868 877 10.1101/gr.9.9.868 10508846 PMC310812

[B46] DeHoon MJL Imoto S Nolan J Miyano S Open source clustering software Bioinformatics 2004 20 1453 1454 LINK http://bioinformatics.oupjournals.org 10.1093/bioinformatics/bth078 14871861

