# Supplementary material for: Transcriptome analyses of early cucumber fruit growth identifies distinct gene modules associated with phases of development
Source: BMC Genomics. 2012 Oct 2;13:518. doi: 10.1186/1471-2164-13-518 (PMC3477022; doi:10.1186/1471-2164-13-518)
Supplement: Additional file 1 — Table S1. Summary of 454 sequencing results and contig assembly for cucumber fruit libraries 0–16 days post pollination. [file 1471-2164-13-518-S1.doc]

Additional File Table S1. Summary of 454 sequencing results (A) and contig assembly (B) for transcript samples of cucumber fruit at 0-16 days post pollination.

_________________________________________________________________

A. 454 Sequencing reads

Sample Clean reads Median Length (bases) Total bases

_________________________________________________________________

0 dpp 251,949 387 90,230,245

4 dpp 235,228 406 88,198,954

8 dpp 187,406 235 41,658,727

12 dpp 234,965 362 84,940,893

16 dpp 221,207 344 76,104,666

Total 1,130,755 381,133,585

_________________________________________________________________

B. Contig assembly

Number of reads assembled into contigs 926,743

Singletons 204,012

% reads assembled into contigs 82.0%

Number of contigs 27,859

Mean contig length (bp) 834

Range of contig length (bp) 52-4,744

Mean number of reads per contig 67

Median number of reads per contig 7

Range of number of reads per contig 2 - 14,522

% contigs with putative homologs in Arabidopsis 64.1%

__________________________________________________________________
