# Supplementary material for: Transcriptome analyses of early cucumber fruit growth identifies distinct gene modules associated with phases of development
Source: BMC Genomics. 2012 Oct 2;13:518. doi: 10.1186/1471-2164-13-518 (PMC3477022; doi:10.1186/1471-2164-13-518)
Supplement: Additional file 2 — Figure S1. Relationship between number of ESTs per contig, mean contig length, and percent of contigs with homologs in Arabidopsis. [file 1471-2164-13-518-S2.ppt]

## Slide 1
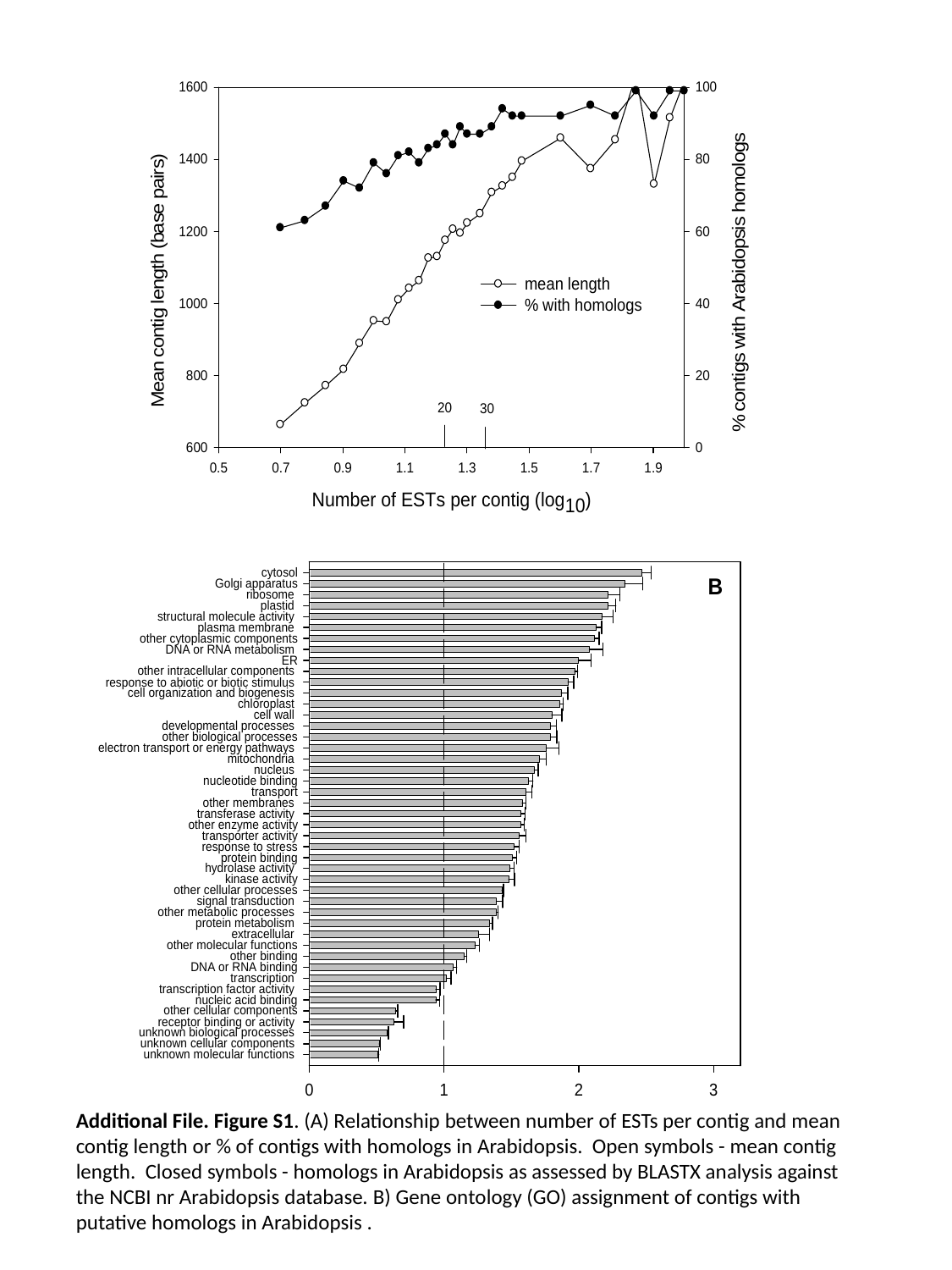

Additional File. Figure S1. (A) Relationship between number of ESTs per contig and mean contig length or % of contigs with homologs in Arabidopsis. Open symbols - mean contig length. Closed symbols - homologs in Arabidopsis as assessed by BLASTX analysis against the NCBI nr Arabidopsis database. B) Gene ontology (GO) assignment of contigs with putative homologs in Arabidopsis .
