# Supplementary material for: Transcriptome analyses of early cucumber fruit growth identifies distinct gene modules associated with phases of development
Source: BMC Genomics. 2012 Oct 2;13:518. doi: 10.1186/1471-2164-13-518 (PMC3477022; doi:10.1186/1471-2164-13-518)
Supplement: Additional file 3 — Figure S2. qRT-PCR verification of gene expression changes. [file 1471-2164-13-518-S3.ppt]

## Slide 1
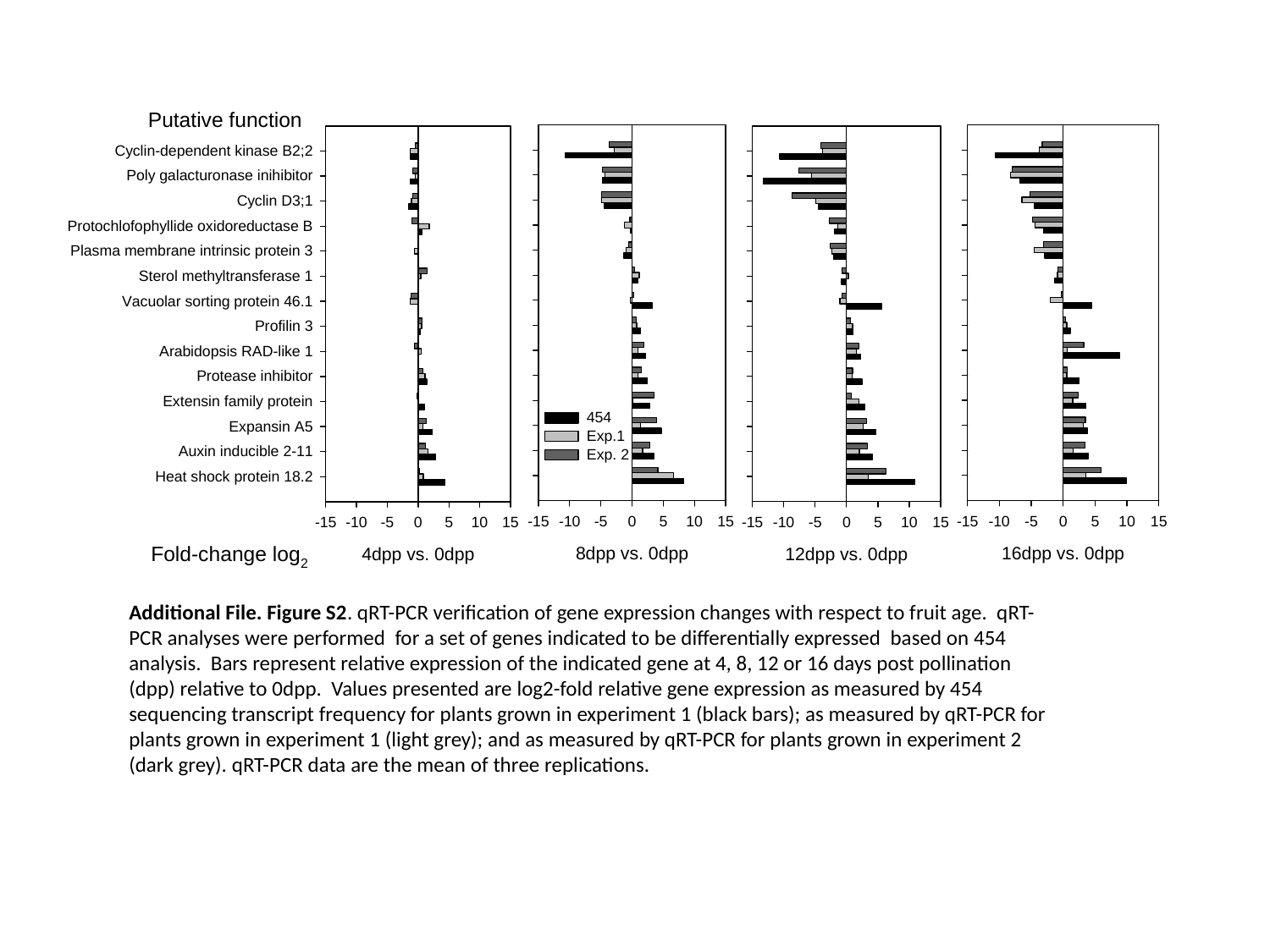

Additional File. Figure S2. qRT-PCR verification of gene expression changes with respect to fruit age. qRT-PCR analyses were performed for a set of genes indicated to be differentially expressed based on 454 analysis. Bars represent relative expression of the indicated gene at 4, 8, 12 or 16 days post pollination (dpp) relative to 0dpp. Values presented are log2-fold relative gene expression as measured by 454 sequencing transcript frequency for plants grown in experiment 1 (black bars); as measured by qRT-PCR for plants grown in experiment 1 (light grey); and as measured by qRT-PCR for plants grown in experiment 2 (dark grey). qRT-PCR data are the mean of three replications.
