# Supplementary material for: Transcriptome analyses of early cucumber fruit growth identifies distinct gene modules associated with phases of development
Source: BMC Genomics. 2012 Oct 2;13:518. doi: 10.1186/1471-2164-13-518 (PMC3477022; doi:10.1186/1471-2164-13-518)
Supplement: Additional file 7 — Table S5. Primers used for qRT-PCR analysis. [file 1471-2164-13-518-S7.docx]

|  | Additional File 7. Table S5. Primers for qRT-PCR | |  |  |
| --- | --- | --- | --- | --- |
|  |  |  |  |  |
|  | Gene | Forward | Reverse |  |
|  | Cyclin-dependent kinase B2;2 | ATATGTGGTCTGTTGCCTGCAT | TCCAGGAAAGAGAGGCTGCTT |  |
|  | Polygalacturonase inhibitor | GGACCAGAGAGACCGTTCCA | ACCTACCATTGCAAAACTCAACAA |  |
|  | Cyclin D3;1 | CCTCACAAACGCAAGCATGA | TGCGTCAATCACGCCATTT |  |
|  | Protocholofophyllide oxidoreductase B | TTAGAGGACTCGCTGGAGGATAA | TCACCACCGTCGATCATTGA |  |
|  | Plasma membrane intrinsic protein 3 | TCTCCGCTACTGATCCCAAGA | CGGAGCTAAAACAGGAATGTGA |  |
|  | Sterolmethyltransferase 1 | TGACCCCTATGTACTTCTTCGTTGT | TGCCCACGTCGTTTTACTCA |  |
|  | Vacuolar sorting protein 46.1 | GAAAGCCATGGAGAAGGGAAA | CCGGATTGCATTCTCAGCAT |  |
|  | Profilin 3 | CCGATAATGGCCGCAGAA | TGAGCATTTGATGTGCGAAATT |  |
|  | Arabidopsis RAD-like 1 | TCATGGGTCAAGTGCATGGA | AAATTGCCAAAGCCTTCTCAAA |  |
|  | Protease inhibitor | TGCTTAGTGGGCTGACAGATGT | TTGGCTTTAATGGCGGTACAA |  |
|  | Extensin family protein | CGCCGCCTCCACCAA | GGCAGAAACGGTGGCAAA |  |
|  | Expansin A5 | GCACGGCGCTTTTCGAT | TGACACAGCGTAGCTCAAAACA |  |
|  | Auxin inducible 2-11 | GACGTTGGGAATCACATCGAT | CAACTTGTGCCTTGGATGGA |  |
|  | Heat shock protein 18.2 | CTCGCATCGACTGGAAGGA | CCGGGAAGATCAGCCTTGA |  |
|  |  |  |  |  |
|  |  |  |  |  |
